# MicroRNA biogenesis and their functions in regulating stem cell potency and differentiation

**DOI:** 10.1186/s12575-016-0037-y

**Published:** 2016-03-09

**Authors:** Shaomian Yao

**Affiliations:** Department of Comparative Biomedical Sciences, School of Veterinary Medicine, Louisiana State University, Baton Rouge, Louisiana 70803 USA

**Keywords:** Stem cells, Differentiation, Cancer stem cells, Cell reprogramming, MicroRNAs (miRNAs)

## Abstract

Stem cells are unspecialized/undifferentiated cells that exist in embryos and adult tissues or can be converted from somatic differentiated cells. Use of stem cells for tissue regeneration and tissue engineering has been a cornerstone of the regenerative medicine. Stem cells are also believed to exist in cancer tissues, namely cancer stem cells (CSCs). Growing evidence suggests that CSCs are the culprit of cancer dormancy, progression and recurrence, and thus have recently received great attention. MicroRNAs (miRNAs) are a group of short, non-coding RNAs that regulate expression of a wide range of genes at a post-transcriptional manner. They are emerging as key regulators of stem cell behaviors. This mini review summarizes the basic biogenesis and mode of actions of miRNAs, recent progress and discoveries of miRNAs in cellular reprogramming, stem cell differentiation and cellular communication, as well as miRNAs in CSCs. Some potential of miRNAs in future biomedical applications and research pertaining to stem cells are briefly discussed.

## Background

MicroRNAs (miRNAs) are short (typically 20–26 nucleotides), non-coding RNAs generated from genomic DNA, and they generally exert negative post-transcriptional regulation (including translational repression, mRNA destabilization, and/or mRNA cleavage) by binding to target mRNAs via the RNA-induced silencing complex (RISC) although upregulation of gene expression by miRNAs has been reported [1, 2]. Studies have revealed that a minimum of 6 bp miRNA:mRNA match (seed sequence) is sufficient to suppress gene expression [3, 4]. Such an imperfect match mode of action allows a given miRNA to target more mRNAs. In general, each miRNA can target many mRNAs (up to more than 100 mRNAs), and multiple miRNAs can regulate a single gene/mRNA [5, 6]. Hence, dysregulating even one miRNA may disrupt the diligent balance of many cellular systems or pathways, which lead to development of diseases or disorders, such as cancers and cardiovascular diseases. Analysis of miRNA expression can find out the problematic miRNAs, which can be the therapeutic targets for curing certain disorders by restoring the dysregulated miRNAs. In addition, miRNAs have been found in many body fluids (blood, urine, milk, saliva, and cerebrospinal fluid), known as circulating miRNAs [7]. These circulating miRNAs (such as miR-21 and miR-181a in human breast milk) are pretty stable and resistant to ribonuclease (RNase) digestion [8]. They may serve as good biomarkers for disease diagnosis [9, 10]. This topic is currently being intensively investigated.

Study of miRNA biology is important on our understanding of the regulation of a wide array of cell functions by miRNAs. Growing evidence shows that miRNAs are a key player in regulating cell differentiation, growth, mobility and apoptosis, and miRNAs have emerged as critical molecular regulators for maintaining the functions of stem cells by fine tuning the protein levels of various factors. Because miRNAs contribute to the specification or differentiation of many cell types, studying miRNAs allows us to understand how miRNAs control the differentiation of stem cells and to elucidate molecular mechanism of cellular differentiation, such as what genes are turned on and off or changing their expression during the differentiation, as well as the molecular networks in controlling cell differentiation. Understanding the regulation of miRNA biogenesis and their mechanism in controlling stem cell differentiation and cell fate determination would facilitate the development of therapeutic approaches in application of stem cells in tissue regeneration and tissue engineering.

### MicroRNA biogenesis and their therapeutic principle

**Fig. 1:**
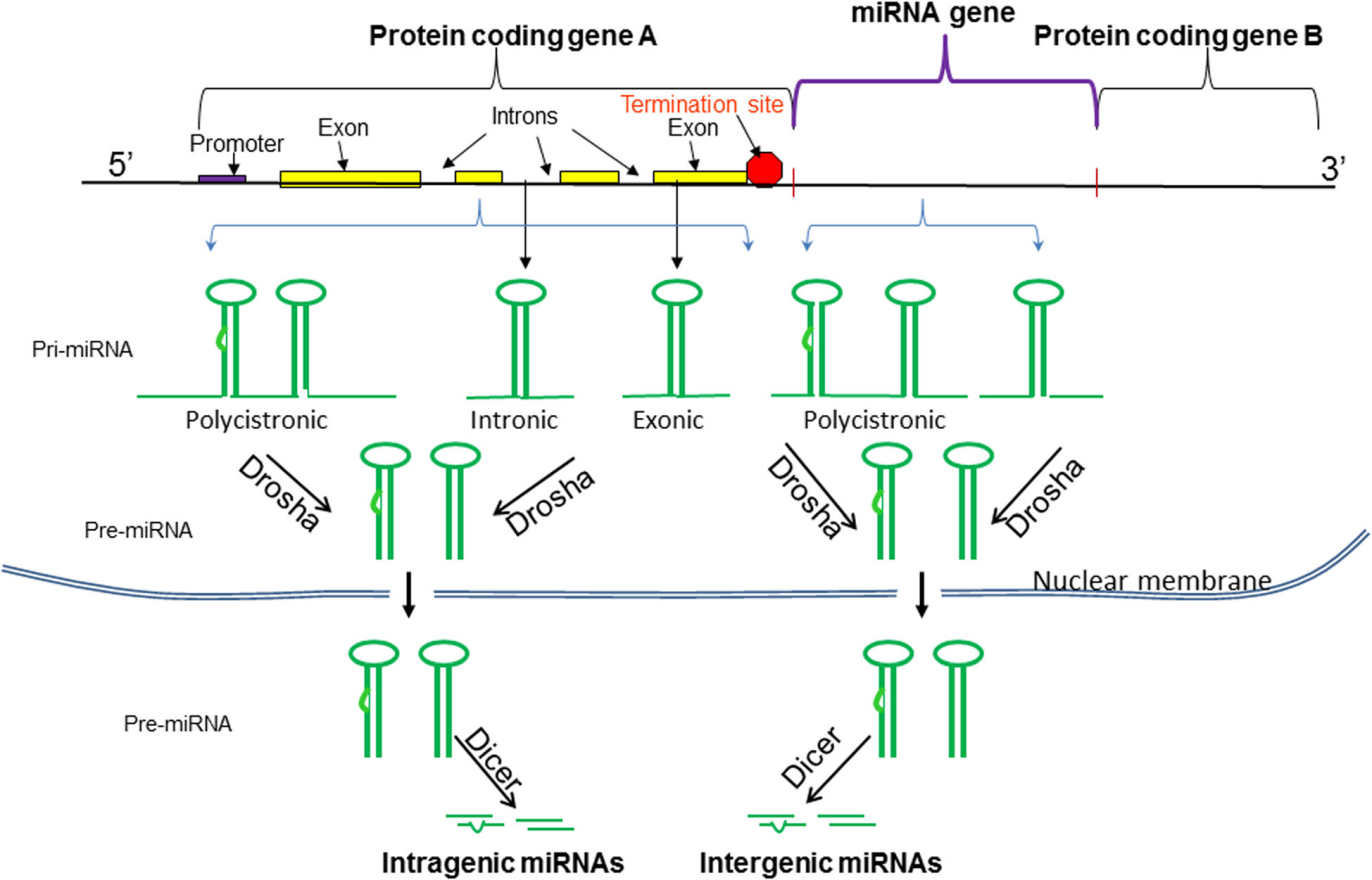
Schematic illustration of microRNA (miRNA) biogenesis from transcription of a protein coding gene and from a miRNA gene. During miRNA biogenesis, a RNA transcript forms stem-loop primary-miRNAs (pri-miRNAs) that can be polycistronic or singular. The pri-miRNAs are processed into hairpin premature-miRNAs (pre-miRNAs) by Drosha. Pre-miRNAs are further cleaved into mature miRNAs by Dicer. Based on their origination, miRNAs can be classified into intragenic miRNAs that are originated from protein coding genes, and intergenic miRNAs that are originated from miRNA genes located between protein coding genes

miRNAs are generated from long double-stranded RNAs. In particular within cells, the miRNA machinery is activated by long double-stranded RNAs to process the RNAs into short miRNAs. Theoretically, any transcript that contains a region of sufficient complementary pairs capable to form a long double-stranded fragment structure can serve as a precursor for miRNAs biogenesis. It should be mentioned that perfect complimentary match is not necessary in the long double stranded region, and few mismatch pairs in the long double-stranded RNA are allowed for activation of the miRNA pathway. Figure 1 illustrates the possibilities of biogenesis of miRNAs in cells. Specifically, miRNAs can be generated by their own genes or by the part of sequences of the protein-coding genes. Primary miRNAs (pri-miRNAs) can be transcribed as polycistronic transcripts (containing multiple hairpin structures in one RNA transcript) or as individual transcripts from intergenic regions, exonic or intronic sequences [11]. On the basis of the location of the miRNA generation, miRNAs are grouped into two classes [12]: (A) Intergenic miRNAs are generated from transcripts of miRNA genes located between protein-coding genes; (B) Intragenic miRNAs are generated from transcripts of sequences located within the protein-coding genes (Fig. 1).

**Fig. 2:**
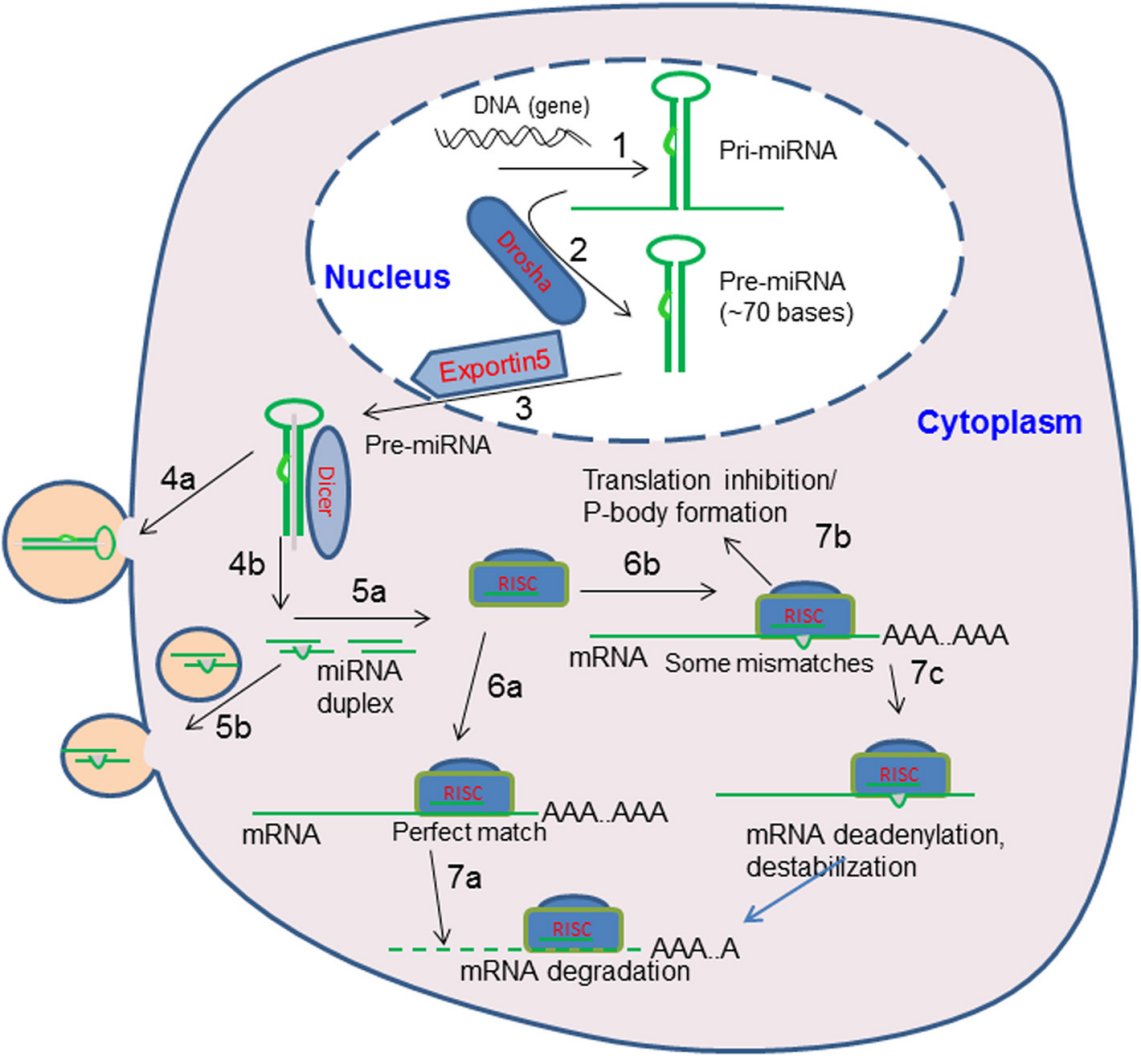
Step-by-step illustration of miRNA pathway in regulating gene expression within a eukaryotic cell: (1) Transcription of DNA into pri-miRNA. (2) Dorsha processes pri-mRNA into pre-miRNA (hairpin shape). (3) Pre-miRNA is transported to cytoplasm by Exportin-5. (4a) pre-miRNAs can be released from the cytoplasm into extracellular environment, or (4b) Pre-miRNA is processed by Dicer to generate mature miRNA duplex in the cytoplasm. (5a) Binding miRNA to proteins to form RNA-induced silencing complex (RISC)*. One of the double strands (i.e., the passenger strand) is removed, and only the guide strand is present, or (5b) secretion of mature miRNAs to outside of the cell. (6a) RISC binds to mRNA with perfect match. (6b) RISC binds to mRNA with some mismatches. (7a) mRNA degradation when the RISC binds to a perfect match mRNA sequence, or (7b,c) either translation inhibition, or mRNA deadenylation leading to mRNA destabilization when some mismatches of mRNA sequence and the RISC. *RISC is also known as miRISC in miRNA-mediated RNA interference pathway

During the miRNA biogenesis, the complimentary RNA sequence is folded into stem and loop structures and then processed to hairpin-shaped premature miRNA (known as pre-miRNA) by a Class 2 ribonuclease III enzyme, Drosha within the nucleus. The hairpin pre-RNA is exported from the nucleus to the cytoplasm by Exportin-5. Next, the pre-miRNA is processed into mature miRNAs by Dicer. Several proteins are then recruited to form RNA induced silencing complex (RISC) with one strand removed and one strand preserved as a guide strand, which can complementarily bind to target mRNAs causing translational repression, mRNA destabilization, and/or mRNA cleavage for post-transcriptional regulation of protein synthesis (Fig. 2). After examining the responses of thousands of genes to miRNAs, Baek et al. concluded that most miRNA target sites are located in the 3′ untranslated regions (3′UTR) of mRNA transcripts [13]. However, other studies demonstrated that miRNA target sites can reside beyond the 3′ UTR in the protein coding sequences (CDS) of mRNA [14]. Some pre-miRNAs and mature miRNAs can be secreted from cells into the extracellular environment (Fig. 2) becoming circulating miRNAs [15] to exert their function/role in other cells within the tissue or in different tissues.

**Fig. 3:**
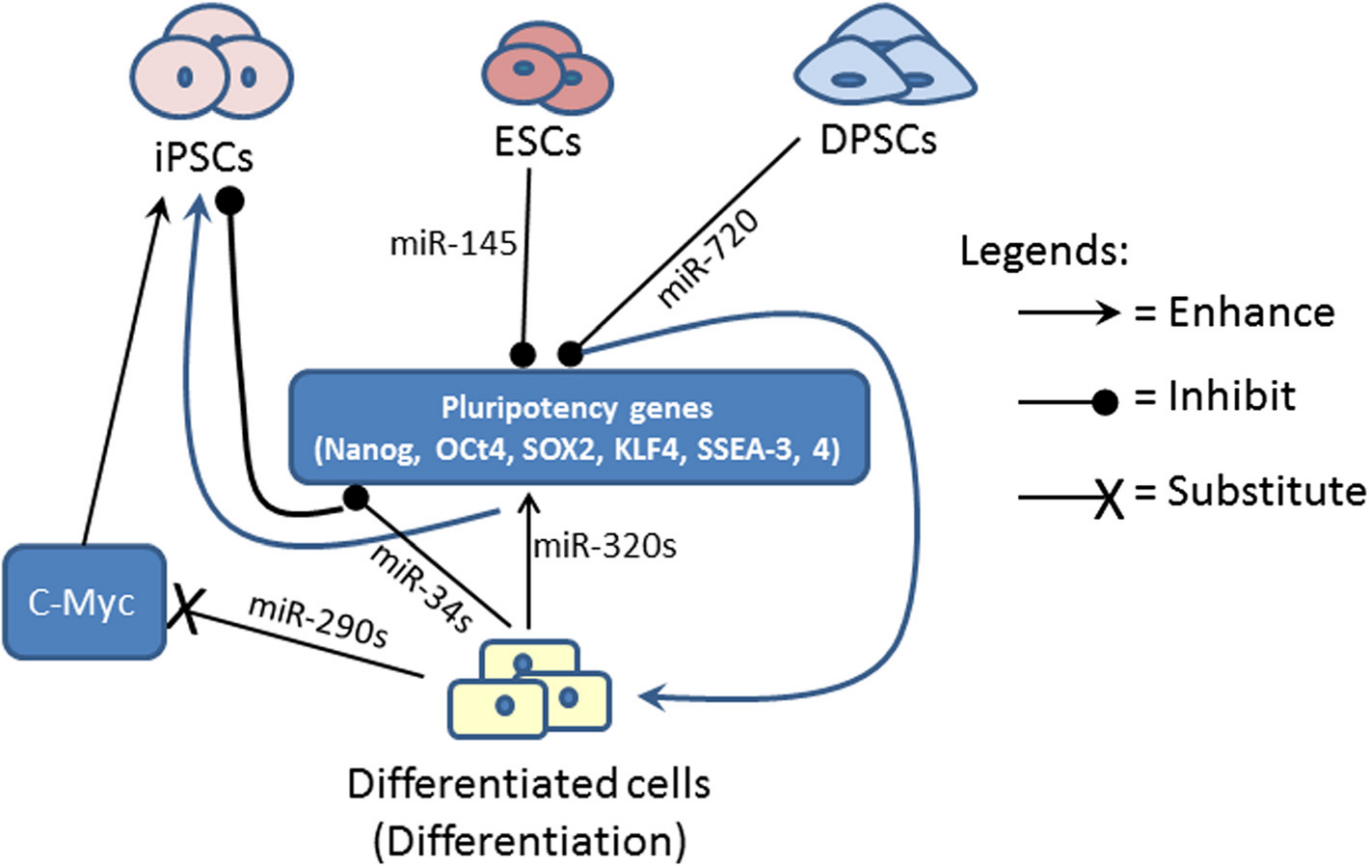
The roles of miRNAs in regulating pluripotency factors in reprogramming differentiated/somatic cells for iPSC production and in stem cell differentiation. In general, miRNAs promote cell reprogramming by enhancing expression of pluripotency factors whereas they enhance stem cell differentiation by inhibiting expression of pluripotency factors

Because of the important role and functions of miRNAs in regulating many aspects of the cellular processes relating to disease development, miRNAs provide new therapeutic targets for drug development in curing many diseases. Generally, miRNA-based therapeutics can be either miRNA antagonists or miRNA mimics. While miRNA antagonists can be used to inhibit the miRNAs that cause diseases, miRNA mimics can be used to increase or restore amount of the miRNAs whose expression is deficient leading to development of the diseases. For example, triple-negative breast cancer (TNBC) is an aggressive subtype of breast cancer. miRNA-profiling studies identified that miR-34a expression was lost in mesenchymal and mesenchymal-stem cell like subtypes of TNBC [16], and lack of miR34a causes significant increased expression of the genes targeted by miR-34a in the cancer cells. Restoration of miR34a expression could significantly decrease proliferation and invasion, and activate senescence of the cancer cells. Currently, miR-34a replacement therapy is being tested in human clinical trials as a promising therapeutic strategy for TNBC [16]. miRNA-based therapies are also considered for treating or preventing infectious diseases. For example, miR-122 is critical for the replication of hepatitis C virus (HCV) [17, 18] by direct interaction with the viral RNA [18]. So miR-122 inhibitors may be prescribed as antiviral intervention treatment for HCV infection in the future [17]. The recent discoveries and breakthroughs in miRNAs indicate that miRNAs may have broad and tremendous impacts in many aspects of biology and medicine. This review summarizes only few important fields of miRNAs that impact stem cell reprogramming, differentiation (Fig. 3, Table 1), and cancer stem cells as well. The emerging field of miRNAs in cell communication is also briefly discussed.

**Table 1:** Functions and roles of miRNAs in stem cell reprogramming and differentiation

microRNAs (miR)	Function	Cell type	Reference
miR-1 and miR-499	Cardiac muscle regeneration	Cardiomyocyte progenitor cells	[39]
miR-15, miR-29, miR-100, miR-133, miR-199, miR-208	Cardiac development		[40] (review)
miR-16, miR-103, miR-107	Inhibit stem cell proliferation	HSCs	[31]
miR-22	Smooth muscle cell (SMC) differentiation	ESC, adventitia stem cells	[37]
miR-26a	Modulated osteoblast differentiation by targeting Smad1 gene	hADSCs	[34, 35]
miR-26a and -26b	Promote adipogenic differentiation	hADSCs	[82]
miR-29	Regulate collagen production for tendon regeneration and remodeling	Tenocytes	[38]
miR-34 s (miR-34a, b, c)	Suppress cell reprogramming	Somatic cells	[25]
miR-125b	Inhibit osteoblast differentiation	mMSC	[33]
miR-128, miR-181	Inhibit stem cell differentiation	HSCs	[31]
miR-134, miR-296, miR-470	Target Nanog, Oct4 (Pou5f1) and Sox2	mESCs	[14]
miR-143, miR-145	Vascular smooth muscle differentiation.	NCSCs	[12]
miR-145	Promote differentiation by downregulating the pluripotency genes, OCT4, SOX2, and KLF4	hESCs	[29]
miR-181	Promote differentiation by targeting Lin28.	HSCs	[83]
miR-200 family	(a) Suppress Sox2 and E2F3 to promote differentiation into neurons. (b) Promote mesenchymal-epithelial transition (MET) to enhance OSKM-induced reprogramming.	(a) Neural progenitors; (b) Fibroblast	(a) [84]; (b) [26]
miR-200c, miR-302 s, miR-369 s	Reprogramming somatic cells to iPSCs	Somatic cells	[22]
miR-290 cluster (miR-291-3p, miR-294, miR-295)	Substitute for c-Myc.	MEFs	[85]
	Increase efficiency of Oct4, Klf4 and Sox2 (OKS) reprogramming		
miR-302s	Conversion of cancer cells to pluripotency.	Cancer cells	[21]
miR-302a	Regulate cell cycle by targeting Cyclin D1	hESCs	[86]
miRNA-302/367 cluster	Reprogram cells into neurons	Fibroblasts	[72]
miR-378	Stem cell survival and vascularization	MSCs	[41]
miR-720	Promote differentiation by repressing the expression of Nanog gene.	DPSCs	[27]

*Abbreviations: DPSCs* dental pulp stem cells, *hADSCs* human adipose tissue-derived stem cells, *HSCs* hematopoietic stem cells, *hESCs* human embryonic stem cells, *mESCs* mouse embryonic stem cells, *MEFs* mouse embryonic fibroblasts, *NCSCs* neural crest stem cells, *MET* mesenchymal-to-epithelial transition, *mMSC* mouse mesenchymal stem cells, *OSKM* OCT4, SOX2, KLF4 and MYC

### MicroRNAs in cell reprogramming

Since the invention of induced pluripotent stem cells (iPSCs) by forced overexpression of the defined transcription factors, intensive studies have been carried out to evaluate therapeutic applications of this technique. One major drawback of the traditional DNA-based reprogramming is the random insertion of the reprogramming factors into the genome in the iPSCs, which could lead to their genome disruption. Extensive research has been conducted to modify the approaches to improve efficiency and safety of the iPSCs using different combinations of transcription factors, delivery methods or using non-genetic approaches, such as using small molecules to induce pluripotency. MicroRNA analysis defined that embryonic stem cells (ESCs) and iPSCs have a distinct miRNA expression pattern as compared to the differentiated somatic cells [19]. This has promoted the research using miRNAs for cellular reprogramming. Human ESCs express abundant miR-302 family, including miR-302a, miR-302a*, miR-302b, miR-302b*, miR-302c, miR-302c* and miR-302d with a highly conserved sequence [20]. Transient transfection of the miR-302s into human cancer cell lines resulted in the conversion of the cells into pluripotent state expressing key ESC markers, such as Oct3/4, SSEA-3, SSEA-4, Sox2 and Nanog (Fig. 3), and having a highly demethylated genome similar to a reprogrammed zygotic genome [21]. Ectopic overexpression of ESC specific miRNAs in somatic cells successfully dedifferentiated the cells into the stem cell stage. For example, Miyoshi et al. reported that a set of three miRNAs (miR-302s, miR-369s and miR-200c) selected from miRNAs that are highly expressed in iPSCs and/ESCs are capable of reprogramming mouse and human somatic cells [22]. The iPSCs induced by miRNAs display similar characteristics as the iPSCs induced by Oct4/Sox2/Klf4/Myc (OSKM). This miRNA-mediated cell reprogramming technique was claimed to be more efficient than the standard OSKM overexpression methods [23]. Because of no concerns of genome integration of miRNAs, miRNA-mediated cell reprogramming may provide an alternative and likely a safer approach for generation of iPSCs as compared to the traditional DNA-based cell reprogramming methods.

The importance of miRNAs in cell reprogramming is also supported by another study demonstrating that the Dicer-knockout fibroblasts (i.e., fibroblasts without mature miRNAs) could not be reprogrammed into iPSCs using the traditional reprogramming factor overexpression method. This suggests that miRNAs are indispensable for cellular reprogramming [24]. While miRNAs facilitating cell reprogramming for generation of iPSCs have been studied, miRNAs that inhibit cell reprogramming were also discovered. It is expected that miRNAs targeting to directly or indirectly reduce the expression of pluripotent genes will suppress or reduce the cellular reprogramming. In this regard, miR-34s (miR-34a, b, c) was found to suppress somatic cell reprogramming by repressing expression of Nanog, Sox2 and N-Myc [25].

The mechanisms of the miRNA-mediated cell reprogramming are not fully understood. It was reported that exogenous Oct4 and Sox2 can bind the promoter regions of miRNA genes to activate the transcription of miR-141/200c and miR-200a/b/429 cluster [26]. Suppression of miR-200 decreased the efficiency of OSKM (OCT4, SOX2, KLF4 and MYC)-induced iPSC generation, whereas forced overexpression of miR-200s using retroviral vector enhanced OSKM reprogramming efficiency by twofold as compared to OSKM only group. Further analysis indicated that miR-200 enhanced OSKM reprogramming efficiency by binding to 3′UTR of the mRNA of zinc finger E-box binding homeobox 2 (ZEB2) to reduce ZEB2 expression [26]. The miRNAs reported to affect cell reprogramming are listed in the Table 1.

### MicroRNAs in stem cell differentiation and tissue regeneration

A side population (SP) is a sub-population of cells sorted with particular markers from a given population. Certain SP, such as Hoechst SP contains high percentages of stem cells. With a locked nucleic acid (LNA)-based miRNA array, miR-720 was found to be highly expressed in the differentiated main population (MP) cells of dental pulp cells as compared to that in the SP cells also derived from the dental pulp cells [27]. Further studies showed that miR-720 represses the expression of the pluripotent determinant gene, Nanog in the dental pulp cells, and repressing Nanog could promote differentiation of dental pulp stem/progenitor cells [27]. In an miRNA profiling study, Hu et al. [28] identified unique subsets of miRNAs that were gradually up- or down-regulated in ESCs during retinal pigment epithelium differentiation, and downregulation of the subset of miRNAs was associated with upregulation of retinal pigment epithelium-specific genes. Xu et al. [29] reported that human embryonic stem cells (hESCs) increase miR-145 expression during differentiation because miR-145 can directly target the 3′UTR of the pluripotency factor genes, OCT4, SOX2, and KLF4 leading to repressing expression of those genes. Downregulation of the expression of the pluripotency genes decreases self-renewal of the ESCs and induces lineage-restricted differentiation (Fig. 3). A long intergenic noncoding RNA, linc-RoR, containing miR-145 binding sites can act as a natural miRNA sponge to sequestrate miR-145. In turn, miR-145 sequestration prevents miR145-mediated downregulation of self-renewal transcription factors Nanog, Oct4, and Sox2 in human ESCs [30].

miRNAs can function in maintaining adult stem cell fate via multiple avenues. In hematopoietic stem cells (HSCs), expression of miR-128 and miR-181 prevents differentiation whereas expression of miR-16, miR-103, and miR-107 inhibits proliferation [31]. miRNAs have been reported to regulate various differentiation pathways including myogenesis, cardiogenesis, neurogenesis, and osteogenesis [32]. One study reported that overexpression of miR-125b inhibited osteoblast differentiation by reducing cell proliferation in mouse mesenchymal stem cells [33]. Bone marrow-derived mesenchymal stem cells (BMSCs) and adipose tissue-derived mesenchymal stem cells (ADSCs) are intensively studied because of their high accessibility. It has been shown that miR-26a is an important miRNA in regulating osteogenic differentiation of BMSCs and ADSCs. In particular, osteogenic differentiation of ADSCs was inhibited because miR-26a repressed the translation of Smad1, a transcription factor required for osteogenesis [34, 35]. Interestingly, miR-26 family was found to increase adipogenic differentiation of human ADSCs by targeting ADAM metallopeptidase domain 17 (ADAM17) [28]. A recent publication reported distinct targeting patterns of miR-26 in BMSCs and ADSCs. Specially, miR-26a mainly targeted on GSK3β to activate Wnt signaling for promoting osteogenic differentiation in BMSCs, whereas in ADSCs, it targeted osteogenic transcription factor Smad1 to suppress BMP signaling to interfere the osteogenic differentiation [35].

miR-22 is a highly-conserved miRNA with multi-functions/roles in epigenetic modification, embryonic development and stem cell differentiation, and it also influences tumorigenesis and development of human diseases/disorders [36]. During smooth muscle cell (SMC) differentiation, miR-22 level is significantly increased. Transfection of miR-22 mimic to overexpress miR-22 in ESCs or adventitia stem/progenitor cells resulted in enhancement of SMC differentiation whereas knockdown of miR-22 by its antagomiR inhibited SMC differentiation, suggesting that miR-22 plays important role in SMC differentiation. Further study indicated that miR-22 exerts such effect via directly targeting and down-regulating Methyl CpG-binding protein 2 (MECP2) expression [37].

The possibility of using miRNAs for improvement of tissue regeneration is currently under investigation. A research team in the University of Glasgow found that miR-29a has potential for tendon regeneration by interacting with interleukin-33 to regulate the production of collagen [38]. Other miRNAs, such as miR-1 and miR-499 were reported to improve differentiation of transplanted stem/progenitor cells for cardiac muscle regeneration [39]. Many other miRNAs, including miR-133, miR-15, miR-29, miR-100, miR-199, miR-208 and miR-378 were reported to be involved in cardiac development, vascularization or cardiomyocyte proliferation [40, 41]. Table 1 summarizes the miRNAs that are involved in stem cell reprogramming and differentiation.

### MicroRNAs in cancer stem cells

Cancer stem cells (CSCs) are believed to be the culprit of the cancer dormancy, initiation and development. Although many sophisticated treatments are available in fighting cancers, survival rate is still pretty low in many cancers. Some proposed that the low survival rate may be due to post-treatment enrichment of the CSCs which is a sub-population of drug resistant tumor cells with abilities of self-renewal, cancer initiation, and further maintenance of tumors [42]. The fact that most malignancies showed dysregulated miRNAs has promoted the effort to study the association between miRNAs and cancers. Numerous recent publications have demonstrated the roles of miRNAs, such as miR-132, miR-182, miR-342 and miR-1225 in cancer cell proliferation, migration, invasion and metastasis [43–47]. Several reviews summarized the association and functions of miRNAs in initiation, progression, metastasis or prognosis of various cancers [48–52]. A searchable database has been established to facilitate the research of cancer-related miRNAs (http://mircancer.ecu.edu/). miRNAs were found to be important in regulating CSCs [53]. This has been demonstrated with osteosarcoma-derived CSCs in a recent publication reporting that overexpression of miRNA let-7d decreased CCND2 and E2F2 cell-cycle-activators and increased p21 and p27 CDK-inhibitors, which cause reduced CSC proliferation. Let-7d overexpression also decreased the expression of pluripotent (or stemness) genes, Oct3/4, Sox2, Nanog, Lin28B and HMGA2 [54].

CSCs can be viewed as generated from the process of dedifferentiation of somatic cells, which is a similar process as cellular reprogramming [55]. Thus, it has been proposed to use miRNAs that can reduce cellular reprogramming to suppress formation of CSCs. CSCs also show dysregulated miRNA expression, targeting such dysregulated miRNAs by miRNA mimics or miRNA inhibitors is currently under investigation for the development of innovative miRNA-based therapy against CSCs [56]. miR-21 was found to be significantly elevated in colorectal cancer, and Deng et al. reported that knockdown of miR-21 could increase the sensitivity of colorectal cancer cells to a classic cancer chemotherapeutic drug, 5-Fluorouracil [57], suggesting that miR-21 is involved in development of drug resistance in cancer chemotherapy. Caudal-type homeobox 1(CDX1) is a transcription factor playing a key role in regulating stem cell differentiation to enterocytes. Loss or reduction of CDX1 expression due to promoter methylation was found in colorectal cancer cell lines [58–60]. In a recent publication, Jones et al. identified that miR-215 transcription is directly targeted by CDX1, and miR-215 then targets to regulate CDX1-downstream genes. Together with other studies, they proposed that miR-215 could offer a novel method to specifically target CSCs [61].

### Cell communications through microRNAs

Cells, either within the same tissue or in different tissues/organs, need to communicate each other to coordinate their behaviors in order to growth, development and survival. Traditionally, cell communication is understood to be accomplished through activation of receptors by a variety of chemical and mechanical signals. Recent studies discovered that miRNAs (e.g., miR-451) can be secreted by various cells and transported to other cells via circulation to affect behaviors of recipient cells in long distance. This provides evidence that cells can communicate in long distance by sending genetic materials/information from one cell to another. Those miRNAs found in circulation (e.g., in plasma) are relatively pretty stable resistant to RNase degradation even under harsh conditions and a long-term (over 10 years) storage, suggesting that those miRNAs are protected by certain mechanisms [10, 62]. The high stability of the extracellular/circulating miRNAs provides the molecules a desirable characteristic for long distance cellular communication via the circulation systems. Several factors are contributing to the stability of those miRNAs, including being packed into exosomes or microvesicles, being loaded into high-density lipoprotein and being bound by AGO2 protein [63–66]. Exosomes, microvesicles, and apoptosis bodies are found in transporting miRNAs for cellular communications. For example, miRNA-containing exosomes are released from mesenchymal stem cells (MSCs) for communications. This includes miR-133b secreted from MSCs and transported to neural cells via exosomes to regulate neurite outgrowth [67], and miR-223 delivered to cardiomyocytes by MSC-derived miR-223 rich exosomes to downregulate Sema3A and Stat3 for cardio-protection in sepsis [68]. For further details in this topic, please see a recent review [69].

### Notions of future miRNA research related to stem cells

Because a minimum of only 6 bp miRNA:mRNA match (seed sequence) is necessary for a miRNA to exert its regulatory function, and in humans, approximately 2000 miRNAs are identified and more novel miRNAs are being discovered, it is expected that miRNAs are involved in regulation of massive number of genes. The importance of miRNAs in stem cell fate determination and differentiation has become nearly ubiquitous. With the progress of the miRNA research, new functions of miRNAs in stem cells will be uncovered and miRNA-based techniques for regenerative medicine may be invented.

Although cell reprogramming with miRNAs was successfully achieved in several reports, there is a controversy whether miRNAs alone can induce the reprogramming or can only improve the efficiency on traditional reprogramming factors [70]. Future studies need to be carried out to clarify the controversy. Furthermore, the mechanism of such miRNA-mediated reprogramming is not fully understood. A recent publication reported that miRNAs likely can modulate N6-methyladenosine (m^6^A) levels of mRNAs by regulating METTL3 methyltransferase binding to mRNAs, and such m^6^A modification can affect cellular reprogramming to pluripotency [71]. Future studies should continue to gain a better understanding of the role of miRNAs in cell reprogramming, as well as in stem cells regulation and activities.

As more novel miRNAs that can be used for cellular reprogramming are discovered and high efficient miRNA delivery methods become available, better methods for cellular reprograming using miRNAs will be established. Pluripotent stem cells have been shown high tumorogenesis after transplantation due to their pluripotency, which is a critical threat for tissue regeneration. Given that the miRNAs are involved in many stages of differentiation, perhaps, a safer approach is to reprogram somatic cells into partially committed stem cells with miRNAs. Such partially committed cells may be multipotent or unipotent, and should be safer than pluripotent iPSCs for regeneration applications. Furthermore, it may directly reprogram the cells into the desired cell types with miRNAs as demonstrated by using miRNA-302/367 cluster and cell-specific miRNAs to reprogram fibroblasts into neurons [72].

It is believed that the circulating miRNAs are involved in cell communications. However, the full functions of the secretory miRNAs are poorly understood. Some studies have shown their pivotal roles in disease development. One of the important aspects in miRNA field is to define the roles of those circulating miRNAs, as well as how to use/control them for therapeutic applications. It is possible to develop methods for efficiently and systematically delivery of miRNAs to control proliferation and differentiation of stem cells for systematic-multiple tissue repairs. This may be done by mimicking stable exosomal miRNAs for delivery of therapeutic miRNAs to regulate or promote proliferation and differentiation of endogenous stem cells for tissue regeneration or repair systematically. In connection with this, exogenous miRNAs from plants or milk were found in the serum and tissues of mice and humans, for instance, the discovery of the rice specific miR-168a in mouse liver to regulate the expression of low-density lipoprotein receptor-associated protein-I [73], implying that miRNAs can be transmitted from dietary sources. Thus, it is possible to formulate drugs or dietary supplements of certain miRNAs to intervene the endogenous stem cells for tissue turnover or tissue repair.

It is known that stem cells can move into wounded or injured sites for repairing the damaged or diseased tissues. Stem cell recruitment into the affected tissues is accomplished through chemoattractants released by the tissues interacting with the chemoattractant receptors on the stem cells. While many chemoattractants have been reported to recruit MSCs [74], stromal cell derived factor-1α (SDF-1α) is the most prominent stem cell homing factor [75, 76]. Release of SDF-1α creates a concentration gradient to promote CXCR4 (SDF-1α receptor)-mediated stem cell mobilization and recruitment [77, 78]. Precise regulation of stem cell movement is critical in maintaining stem cell homeostasis within tissues. It was reported that miRNAs can regulate expression of SDF-1α (by miR-27b, miR-126, miR-126*) or CXCR4 (by miR-150), and thus miRNAs participate in regulation of stem cell recruitment [79–81]. Given that circulating miRNAs are stable and can function in cell communications, it would be interested to study if miRNAs can directly serve as signals to recruit stem cells to the injured or diseased tissues.

Because one miRNA can regulate expression of many genes, miRNAs are easily interwoven into regulatory networks in controlling cell fate and differentiation. Given that the function of circulating miRNAs in cell communication, miRNAs may not only involve in crosstalk between different molecular pathways within a stem cell or stem cells within a tissue, but also participate in crosstalk of the pathways in tissue-derived stem cells in different tissues or organs. This will become clear as the progress of miRNA research in the future.

## Conclusion

In conclusion, miRNAs are short non-coding RNAs generated from transcripts of genomic DNA. A minimal of 6 bp seed sequence of miRNA:mRNA match is necessary to activate RNA interference pathways to exert post-transcriptional regulation, and such imperfect match mode of action of miRNAs allows one miRNA to regulate many genes and many genes to be regulated by one miRNA. Thus, it would not be surprised to see that miRNAs are critical in regulating diverse aspects of stem cells. miRNA has become an important field of biology and biomedical research including improvement of miRNA detection, therapautic application of miRNAs and studying the mechanisms of miRNA regulation. With the progress of the miRNA research, cutting-edge methodology in applications of miRNAs will be developed in cell reprogramming for generation of iPSCs and in controlling stem cell proliferation and fates for tissue regeneration and tissue engineering, as well as development of miRNA based methods for cancer prevention and treatment.
